# On Abdominal or Ileo Typhus

**Published:** 1871

**Authors:** Carl Proegler

**Affiliations:** Aurora, Illinois


ARTICLE II.—*On Abdominal or Ileo Typhus.* By CARL PROEGLER, M.D., Aurora, Illinois.

During the siege of Metz, we had quite a number of typhus cases, and I shall try to give my experience in the treatment, etc. I shall not quote the personal cases out of my diary, but give the statement in extenso.

I had over seventy cases under my special charge, twenty-eight of which were very hard cases, twenty medium cases, and the rest of twenty cases we may class under the head of febriculeuse typhus; they generally kept along for three weeks, some even could get discharged from medical treatment within a fortnight.

The whole picture of ileo typhus is so much known, that I scarcely deem it necessary to give a minute symptomatology of the cases which occurred in my wards. But every epidemic has its peculiarities, and it is these which I shall describe.

The beginning of sickness commences with higher temperature of the skin and a great rigidity of the patient experienced in all the limbs; not a feeling of fatigue or lameness, but certain muscular contractions were predominant.

Especially the muscles of the neck and back, which could not very properly be controlled by the patient; the latter did not seem to care much, and after admittance to the hospital, would like to creep about. They all did not like to lie down, and they kept sometimes six to eight days in that condition without coming under the notice of the doctor. No appetite. The tongue became to appearance alike with all the patients. The edges pale red; the interior of the tongue was covered with a yellow gray or gray coating. The tongue moist; but it cost the patient quite an exertion to show it, and then with tremulous movement.

The expression of the countenance was languid, the eyes lost their lustre, the cheeks redder than usual. By-and-by the head grew heavy and dizzy, preventing the patients walking or going, and only these symptoms would bring them to bed.

In only four cases I noticed headache, being then the only complaint. Sometimes nose bleeding occurred, but not very often or great. Exploration at that time revealed a tumate spleen, sometimes so great that the spleen could palpably be percussed.

Abdomen not very much enlarged, and even by great pressure not very painful; and, strange, the ileo cœcal was painful with only a few. On the contrary, the patients would experience, even with slight pressure, great pain in the regio epigastrica, and around the spleen. Gargouillement was noticed many times, especially in cases with diarrhœa. The latter would not occur oftener than two or three times daily.

The feces were thin, of a dark brown yellow color, not profuse, and of pungent odor.

The pulse was, in intense cases—which I am now especially noticing—very different, ranging from 96 to 128. One very aggravated case, resulting in death, was characterized by a persistent pulse of 104.

With these symptoms the ileo typhus ripens into full bloom—dizziness greater, the expression became more apathetic, with slight delirium.

I have seen quite a good number admitted in these stages, who had done their duty up to the time, but as soon as admitted, or very shortly afterwards, all the symptoms of aggravated typhus made their appearance. Some would sigh unconsciously. One very intense case, with very early and aggravated symptoms of collapse, —where the patients for full six weeks were groaning very loud—with every expiration, like one deeply affected, sighed deeply.

I did not notice much roseola. My patients having been mostly soldiers from the rural districts, and not used to a great cultivation of the skin, it might have been that they were not early visible. Only in a few cases I could detect five or six roseola patches as large as a fleabite.

The hearing of the patients got impaired from day to day, ending in almost complete deafness, even in patients where the sensorium was perfectly, or in a measure clear, and who were only seemingly languid and fatigued. I was obliged to talk to them very loud to make myself understood, showing that deafness in ileo typhus is a nervous symptom. The patients did not complain of anything except occasional vertigo, especially when I was obliged, for a physical exploration of the lungs, to have them sit up. The tongue got more and more dry, cracked and beefy; only the edges had a curious dark red color, and looked as if they were varnished. I saw only twice a tongue red and varnished without any coating.

The dryness of the tongue is always a good measure of the mental state of the patient; seemingly they do not know that they take too little fluid, or if they know, then they have no will to give expression to the thought and desire for fluids. Both I have noticed, and it seems justifiable to presume that the dryness of tongue has something to do with the mental derangement.

Diarrhœal discharges, like those noticed before, occurred about five or six times every twenty-four hours, and even oftener. In some cases constipation was prevalent, so that castor oil had to be employed.

Nose bleeding occurred often, and I could not help noticing that the patients after every nosebleed had a better expression, and that the sensorium became brighter. Only in two cases I noticed that the patients became paler.

Hemorrhage from the bowels I noticed in eight; all of them had an enlargement of the spleen. The same I noticed in a colleague of mine. He had a very large spleen, and perished by exhaustive nose and intestinal bleeding.

How both these symptoms were connected together, I do not dare to explain. But if future cases should develop an affinity between nasal and rectal bleeding, it would not be impossible that the changes of the blood by anatomical changes of the spleen would lead on the other side to *anomalous changes of the nutrient blood vessels*, so that they may be more easily torn. Bleeding appeared in two ways, either mixed together with the stools, giving the latter a rather chocolate color, or in thick, dark colored clots. It is very probable that the blood, especially in the last cases, comes from the lower part of the intestines. In one case I noticed, together with this hemorrhage, copious diarrhœa, coming so often that the entire bed was always drenched with the dejections. The third week showed a marked decrepitude of the patients, which I hardly ever witnessed in hospital practice before.

The paniculus adiposus went off with rapidity; the patients were pale, emaciated, half or wholly comatose. The countenance haggard and wrinkled, without any expression; they seemed to represent a picture of total indifference. The extremities inclined to coldness; in some cases the feet were entirely cold. Dicrotic pulse; 120 beats and above; the art. radialis not much flexed, somewhat wider; the pulse wave not very high; the sounds of the heart were dull, especially the first sound, which was cracking and hardly to be heard. It is not without intention that I describe these symptoms more closely; they gave me the basis for the therapeutic treatment, and showed that nothing is more foolish at the sick bed than to be guided by some one-side principles concerning therapeutical treatment in one and the same sickness, especially when the same develops itself in individuals who are totally different concerning their mode of living, occupation, etc. Only with the greatest of care did I succeed in warding off collapses. At the same time with the other symptoms the patients had bronchial catarrh, much coughing and tenacious sputa, tormenting the patients a good deal. Thickening of the parenchyma of the lungs in the lower parts, with a dull sound and difficult breathing; only twice I noticed lobular pneumonia. Bed sores I noticed in three cases, and not sooner than the third week; they occurred at the os coccygis, the back, and even the buttock. In two cases the sores were light; in one case the sores reached the bone, with a good deal of loss of substance.

With but few exceptions the delirium was of a mild character; sometimes a patient got out of bed and wanted to go off, but was very easily persuaded to go back to bed. There seemed to be no fixed ideas with any of the patients. It was strange how generally contented they were; if asked how they felt, they would always reply in a kind of careless manner, as though thinking others and not themselves to be affected by sickness.

On account of sickness among the employes of the hospital, I had not always sufficient assistance and could not make thermometrical observations, therefore I endeavored to discover a symptom that could quickly be discerned and that could be taken as the forerunner of recovery.

The careful physician will never depend on single symptoms, because he may be misled by every one of them. The complex of symptoms, and their relation to each other, assure diagnosis and prognosis. The lowering of temperature may not be an indication of recovery; on the contrary, it might be rather ominous; so with the pulse, but combined together it will give a pretty good test for the patient. I only say this that I may not be accused of having followed blindly single symptoms alone.

It cannot be denied that one symptom may be worth more attention than another; and after a careful observation I noticed that in typhus the appearance of the tongue will be in some measure an indication for a change for the better with the patient.

The symptomatology of the tongue is, like a good many important signs held highly by the older physicians, not now considered of great importance. The exact physico-chemical researches made sad havoc with those merely superficial observations of the old writers of medicine, and the medical art has won generally, but not in every respect. The greater number of our modern doctors who based on physical exploration, have lost sense for the simple, unostentatious and careful observations of the sick. The physico-chemical researches come in the first line, and it being true that the diagnosis has been made sure by help of those means, there are remaining certain things, which cannot physically be proved, but which are highly important for the physician to know. Sometimes one symptom, sometimes the totality of symptoms, will be more useful for therapeutics than the proof that the limits of a pleuritic exudation have increased half an inch, the proof being in the first line important. Especially in typhus there are so many symptoms which cannot be physically, nor by the means of auscultation or percussion, nor with the help of the thermometer, explained; and I have found that the tongue in typhus was a great help to me concerning prognosis and therapeutics. The patient may feel easier in his head, the pulse may become slower, the temperature lower; all these symptoms explain the exact period in which the patient is, but are not an exact indication of the state of the patient, because sudden changes are not very seldom noticed, and the patient remains just so long in danger till the tongue has assumed quite another appearance. The signs of approaching recovery are the following:

The cracked and heavily coated tongue which is dry and sometimes immovable, so that the patient only with an effort can show it, becomes moist and has a slippery touch; the cracks loosen; and what with the extremest cleanliness by washing, etc., could not be effected, nature brings about in a few weeks.

The edges of the tongue get pale red and the middle part whitish gray; the tongue protrudes easy and does not tremble. After a little while the patient experiences some appetite, assuming by and by the character of hunger; the countenence loses its careless habitus, and the patient will now look very pale and emaciated; the eye is bright; head free; sleep quiet and long, firm; stools only once a day and thicker, with quite a normal appearance; the cough gets looser, respiration slower, and expectoration more abundant. The arteries are but slightly flexed, the artery itself narrow; pulse quiet, but rises with the slightest exertion to 120. With favorable circumstances the patient gets into the state of convalescence. In only four cases have I seen a change for the worse after the patient has been in the last described state; two went into regular typhus collapse; the fourth was taken with hemorrhage again, and I could trace his relapse to an error in diet, having received from some one a good deal of cake, and was lying for weeks almost on the point of death; he had bed sores. In the other three cases no errors in diet could be traceable. The re-beginning of the sickness commences with the coating of the tongue, dryness, etc., hot skin, tumor of the spleen, and some roseola. All four cases recovered after a sickness of four months' duration. They were respectively 20 years of age, robust and healthy individuals.

The mortality was about 16.60 per cent.

There has been so much written on the treatment of typhus, that I hardly hope to offer anything new. Only one point I consider of importance. Medicine is and ought to be remembered as natural science in the fullest sense of the word. Taking it in this sense, it requires, firstly, a precise and unbiassed study of nature; and, secondly, to try to find out by all possible means the way of nature. This must not be only a saying with professional men, but it must be truth, hence it has been with all great physicians of all ages; and there is nothing more foolish than to think, that nature can be forced to point out and shape the healing process. I do not want to be understood that I preach *nihilismus* which compares the doctor at the sick bed to an actor, but only the pressing necessities to individualize and diagnosticate every new case according to the indications. Only this point of view will help therapeutics, and only treated in this sense, it may compare favorably with the teachings of diagnosis and prognosis.

Typhus and the treatment of it has led to these remarks, because there is already a tendency to take a single symptom for the whole disease, neglecting, therefore, the individual, and treating it empirically.

Liebermeister, Hagenbach, Jurgensen, and a good many others, are of opinion that by reducing the fever by heroic means typhus will be less dangerous. The results obtained by those authors are unquestionably great, and the treatment ought to convince us of its efficacy. I had already ample opportunity to study in the different clinics, both here and in Europe, the different treatment, and was struck with the great success Traube met in the Berlin Charite Hospital. He did not use cold bathing, but only cold ablutions. Although the cooling off was not as great as by bathing, still the temperature of the body was diminished. There was general improvement—head clearer, pulse slower and fuller, expression more marked, etc.

These observations led me to adopt the same plan of treatment in a military hospital where there being such constant change of nurses I could not use the cold bath and the wet sheet. I had only two occasions to test it. I used either compresses or flexible ice bladders, changing them every five or ten minutes. They were dipped in ice water, and the patient was covered on his chest, abdomen and head with it; having detailed for that purpose quite a number of soldier-nurses. The success I met with was not very great, the patient getting lower in the second and third week, and especially the extremities became cold, the pulse slow and easy, countenance pinched and shallow, so that I had to change. The most aggravated cases showed the symptoms first. Was it that cold compresses are a bad substitute for the hydropathic treatment, or could my patients not stand cold?

Liebermeister asserts, as his observation, that moderate loss of warmth in health and fever will not cause internally the same change, but on the contrary it will give rise to a higher temperature. And only in that way can I explain the effects of my compresses. Covering only the sick part of the body, they took away only a small amount of warmth, leaving the sick process almost unchanged. For the truth of this observation I can vouch, and I think it, therefore, better not to use the hydropathic treatment if it cannot be properly applied. But certainly that will be a debatable point; it may be that those individuals could not stand the hydropathic treatment. It must be remembered that they were soldiers and mostly French, who had endured a good deal of hardship and privation, and probably had not enough vitality in the system. It was not so much an object to suppress the fever as to feed them as much as possible.

*Ex juvantibus*, I draw my conclusion.

I used in the beginning: acid hydrochl., (2, 5 : 200,) and chinin, (0, 3) every two hours. For nourishment they had milk, beef tea, (but I had to change soon to strong beef tea), yolk of eggs, wine, etc., especially port and sherry wine. Chinin in small doses was continually used, and I made the observation that small doses of chinin perhaps (0, 6 : 200,) every two hours a tablespoonful, produced a tonic effect, larger doses producing less activity of the heart, and being, therefore, really anti-febrile. The pulse gets slower, even irregular, the radialis loses its flexibility, and the waves gain in height as shown by the sphygmograph. Strong stimulating remedies I used only in collapse, and especially acid benzoic (0, 12), and g. camphor, (0, 06), every two hours one powder.

In two cases I saw quite a remarkable change, patients being almost gone. We did not use musk, on account of its high price, but Traube recommends it highly. In one case only, the repeated use of musk saved the patient (the only case where musk was used), but it should be considered whether diarrhœa is present or not, as the benzoic acid would not do in the latter complication. The quintessence of the treatment consisted in good nourishing diet and heavy wines.

Hemorrhage of the bowels ought to be very carefully watched, and I can recommend highly the liquor ferri sesquichlorid. I gave it very often, every two hours in water, (5 gtt.) The bleeding stopped, and even when it occurred again it had the same effect.

For the diarrhœa I used ext. senna, strych. spirit, (0, 12 : 200), every two hours a tablespoonful, but always given with mucilaginous drinks.

For bedsores I used emplast. saponat., spread on deer skin, and permanganate of potass. (1, 3 : 120.)

